# Commentary: Cafeteria diet impairs expression of sensory-specific satiety and stimulus-outcome learning

**DOI:** 10.3389/fpsyg.2015.00536

**Published:** 2015-04-30

**Authors:** Shauna L. Parkes, Teri M. Furlong, Fabien Naneix

**Affiliations:** ^1^Institut National de la Recherche Agronomique, UMR 1286, Nutrition and Integrative NeurobiologyBordeaux, France; ^2^Centre National de la Recherche Scientifique, UMR 5287, Institut de Neurosciences Cognitives et Intégratives d'AquitaineBordeaux, France; ^3^Université de Bordeaux, UMR 1286 and UMR 5287Bordeaux, France; ^4^Department of Neurobiology and Anatomy, University of Utah School of MedicineSalt Lake City, UT, USA

**Keywords:** diet, sensory-specific satiety, Pavlovian, decision making, obesity

Current global estimates indicate that the proportion of adults meeting the criterion for overweight and obesity is 40%, with this proportion expected to increase (Ng et al., 2014). Thus, diet is arguably the largest controllable factor related to the burden of disease, yet changing dietary habits is notoriously difficult (Caballero, 2007) and the reason for this is unknown. Mounting evidence suggests that, in addition to contributing to the unprecedented rates of obesity worldwide (Caballero, 2007), the consumption of high fat, high sugar (HFHS) diets is associated with a range of cognitive impairments in humans (Smith et al., 2011; Gustafon et al., 2012) and non-human animals (Beilharz et al., 2014; Reichelt et al., 2015). Such data raise the possibility that intake of calorically dense foods may alter cognitive capacities critical for food-related decision making and, as a result, make it more difficult for individuals to change their eating behaviors.

In a recent issue of Frontiers, Reichelt et al. (2014) examined the effect of a highly palatable and caloric rich (“cafeteria”) diet on food-related cognition in rodents. Specifically, they used Pavlovian devaluation to determine if consumption of a cafeteria diet affects the ability to learn about food-related stimuli. Rats were trained to associate two cues with two distinct foods after which one of the foods was devalued via sensory-specific satiety, defined as a rejection of a food recently eaten to satiety while readily consuming another food with distinct sensory properties (Rolls, 1986). Rats fed a standard chow diet responded less during the stimulus that predicted the devalued food than during the stimulus that predicted the still valued food. In contrast, cafeteria-fed rats responded equally during both stimuli. Based on this result, the authors conclude that consumption of the cafeteria diet produces a deficit in the expression of stimulus-outcome learning and, in particular, of cue-food associations.

However, in addition to the deficit observed in Pavlovian devaluation, the authors also reported that, following satiety-induced devaluation, cafeteria-fed rats failed to show sensory-specific satiety and consumed equal amounts of the devalued and valued foods. Indeed, following selective satiation, cafeteria-fed rats reduced their consumption of both the devalued and valued foods. As such, the authors have provided clear evidence that satiety-induced devaluation was not effective in selectively reducing the value of the prefed food in the cafeteria-fed rats. The reliance on this devaluation treatment in the Pavlovian task therefore poses a considerable confounding factor. Specifically, the deficit observed may not reflect impaired stimulus-outcome learning, as suggested by the authors, but instead could be attributable to the cafeteria-fed rats' insensitivity to selective satiety-induced devaluation. To assess if consumption of a cafeteria diet impairs the ability to learn and express cue-food associations a task that can selectively devalue the prefed food should be used, for example, lithium chloride-induced devaluation (Holland and Straub, 1979; Singh et al., 2010). Alternatively, a Pavlovian-instrumental transfer task could be used to evaluate the capacity of a food-related stimulus to invigorate responding for a specific food (Corbit and Balleine, 2003). Both of these procedures allow the assessment of stimulus-outcome associations in cafeteria-fed rats independently of the observed deficit in sensory-specific satiety.

Using a similar devaluation procedure, Furlong et al. (2014) recently reported that consumption of a HFHS diet promotes habitual food-seeking. However, in contrast to Reichelt et al, Furlong and colleagues reported no effect of the diet on sensory-specific satiety. Rats with and without a history of a HFHS diet consumed more of the valued than the devalued food when the foods were freely available, indicating that altered food-seeking behavior was not secondary to compromised specific satiety and was instead due to altered learning. The difference in sensitivity to specific satiety-induced devaluation reported by the two studies is striking. While there were a number of small procedural differences (e.g., choice vs. non-choice of the valued and devalued food, time between devaluation and the sensory-specific satiety test), there was also an important difference in the accessibility of the high calorie diets. Specifically, Reichelt et al. used continuous access whereas Furlong and colleagues used intermittent access. It is not immediately clear why continuous versus intermittent access would differentially affect sensory-specific satiety however, it is reasonable to speculate that animals with continual access to a high calorie diet may become less sensitive to the immediate sensory impact of food. Studies examining the adaptation of sensory systems indicate that repeated exposure to the same stimulus results in an attenuated neural response to that stimulus and a diminished perceptual experience (Clifford et al., 2007; Webster, 2012). Indeed, chronic exposure to a high calorie diet induces a decrease in both the consumption of palatable foods (Duca et al., 2014) and in hedonic reactions to these foods (Shin et al., 2011). Moreover, a number of studies have reported differential effects on cognition depending on the nature of the access to the diet (Colantuoni et al., 2002; Avena et al., 2005; Furlong et al., 2014; Martire et al., 2014). For example, intermittent, but not chronic, access to a high calorie diet promotes habitual food-seeking (Furlong et al., 2014) and binge-like eating patterns (Martire et al., 2014). Given these differences in accessibility of high calorie foods it cannot be assumed that the diet used by Reichelt and colleagues would necessarily result in deficits in the learning and expression of food-cue associations, but the question warrants further investigation.

Increasing evidence suggests that consumption of calorically rich foods leads to changes in cognitive control which makes subsequent changes to eating behaviors more difficult. Research into the nature of these cognitive deficits is therefore highly valuable for instantiating changes in eating behaviors to combat the obesity epidemic. Reichelt and colleagues have provided important evidence that continuous exposure to a Western-style diet disrupts sensory-specific satiety, an effect that, in humans, may result in over-consumption of food (Hetherington, 1996). However, it still remains to be determined if such diets also impair learning about food-related cues, a result that has more far-reaching consequences for effective dieting strategies and decision making in general (Balleine and O'Doherty, 2010).

## Conflict of interest statement

The authors declare that the research was conducted in the absence of any commercial or financial relationships that could be construed as a potential conflict of interest.
